# Triglycerides in the Human Kidney Cortex: Relationship with Body Size

**DOI:** 10.1371/journal.pone.0101285

**Published:** 2014-08-29

**Authors:** Ion Alexandru Bobulescu, Yair Lotan, Jianning Zhang, Tara R. Rosenthal, John T. Rogers, Beverley Adams-Huet, Khashayar Sakhaee, Orson W. Moe

**Affiliations:** 1 Department of Internal Medicine and the Charles and Jane Pak Center for Mineral Metabolism and Clinical Research, University of Texas Southwestern Medical Center, Dallas, Texas, United States of America; 2 Department of Urology, University of Texas Southwestern Medical Center, Dallas, Texas, United States of America; 3 Department of Internal Medicine, University of Texas Southwestern Medical Center, Dallas, Texas, United States of America; 4 Department of Clinical Sciences and the Charles and Jane Pak Center for Mineral Metabolism and Clinical Research, University of Texas Southwestern Medical Center, Dallas, Texas, United States of America; 5 Departments of Internal Medicine, Physiology, and the Charles and Jane Pak Center for Mineral Metabolism and Clinical Research, University of Texas Southwestern Medical Center, Dallas, Texas, United States of America; University of Milan, Italy

## Abstract

Obesity is associated with increased risk for kidney disease and uric acid nephrolithiasis, but the pathophysiological mechanisms underpinning these associations are incompletely understood. Animal experiments have suggested that renal lipid accumulation and lipotoxicity may play a role, but whether lipid accumulation occurs in humans with increasing body mass index (BMI) is unknown. The association between obesity and abnormal triglyceride accumulation in non-adipose tissues (steatosis) has been described in the liver, heart, skeletal muscle and pancreas, but not in the human kidney. We used a quantitative biochemical assay to quantify triglyceride in normal kidney cortex samples from 54 patients undergoing nephrectomy for localized renal cell carcinoma. In subsets of the study population we evaluated the localization of lipid droplets by Oil Red O staining and measured 16 common ceramide species by mass spectrometry. There was a positive correlation between kidney cortex trigyceride content and BMI (Spearman R = 0.27, P = 0.04). Lipid droplets detectable by optical microscopy had a sporadic distribution but were generally more prevalent in individuals with higher BMI, with predominant localization in proximal tubule cells and to a lesser extent in glomeruli. Total ceramide content was inversely correlated with triglycerides. We postulate that obesity is associated with abnormal triglyceride accumulation (steatosis) in the human kidney. In turn, steatosis and lipotoxicity may contribute to the pathogenesis of obesity-associated kidney disease and nephrolithiasis.

## Introduction

The prevalence of obesity (body mass index ≥30) among adults in the European Union ranges from 8% in Romania to 24% in the United Kingdom [1], exceeds 35% in the United States [2], and is approximately 11% worldwide [3]. Although body mass index (BMI) is not an ideal measure of body adiposity and associated health risk [4], multiple studies have shown that increased BMI is an independent risk factor for chronic kidney disease (CKD) and end-stage renal disease (ESRD), even after adjustment for obesity-related conditions such as hypertension and type 2 diabetes [5]–[7]. The mechanisms by which obesity can directly contribute to increased CKD and ESRD risk, independent of its association with hypertension and type 2 diabetes, are incompletely understood. Various non-mutually exclusive and partly overlapping mechanisms have been proposed, including inflammation, hyperfiltration, podocyte stress, oxidative stress, changes in various hormones or signaling molecules such as leptin and adiponectin, as well as renal lipid accumulation and lipotoxicity [8]–[11].

In addition to its association with CKD and ESRD, obesity has been linked with increased risk for kidney stones in general [12], [13], and uric acid stones in particular [14], [15]. While the pathophysiology of uric acid nephrolithiasis is likely multifactorial [16], animal and cell culture experiments have shown that lipid accumulation in proximal tubule cells may contribute to the urinary biochemical abnormalities that underpin uric acid stone risk [17]–[19].

Lipid accumulation in other organs, including skeletal muscle, myocardium, pancreas and liver, has been associated with obesity in humans [20]–[23], and has been implicated in cell and organ dysfunction [24]–[27]. Lipid accumulation in the kidney has been described in a number of animal models, but very little human data are available [8], [9]. In particular, establishing whether renal lipid accumulation occurs in humans with increased BMI, thus potentially contributing to obesity-related CKD, ESRD and nephrolithiasis risk, is of fundamental importance, and there is no database on this topic. To address this knowledge gap, we measured renal triglycerides and defined their localization in normal kidney surgical specimens obtained from patients undergoing nephrectomy, with a wide range of BMI. In addition, we measured tissue levels of 16 common ceramide species in representative samples.

## Methods

### Ethics Statement

The human study was approved by the University of Texas Southwestern Medical Center Institutional Review Board, was conducted in strict accordance with the Helsinki Declaration of 1975, as revised in 2000, and all study participants provided written informed consent prior to nephrectomy. The animal study protocol was approved by the Institutional Animal Care and Use Committee (IACUC) at University of Texas Southwestern Medical Center, and all animal work was performed in strict accordance with institutional guidelines and with the National Academy of Sciences Guide for the Care and Use of Laboratory Animals.

### Study participants and tissue collection protocol

We studied surgical specimens obtained from patients undergoing total nephrectomy at the University of Texas Southwestern Medical Center and affiliated hospitals between 2007 and 2012. Included in the study were patients older than 21 years, of either gender and of any race/ethnicity, undergoing nephrectomy as first-line therapy for unilateral renal cell carcinoma. Race/ethnicity was self-reported. Exclusion criteria were serum creatinine ≥1.5 mg/dL, proteinuria, treatment with insulin or thiazolidinediones, genetic diseases of the kidney, inborn defects of lipid metabolism, and a history of recurrent urinary tract infections. None of the study participants were alcohol abusers. After nephrectomy, an experienced surgical pathologist dissected 1–3 separate, 0.1–1 cm^3^ normal kidney cortex samples from each kidney, away from the tumor. Patients with no normal kidney cortex upon pathological examination of the surgical specimen were excluded from the study. Tissue samples were immediately frozen in liquid nitrogen and stored at −80°C for biochemical measurement of triglycerides. For 29 subjects from which 2 or more samples were available, one sample was fixed in 4% paraformaldehyde, cryosectioned and stained for lipids using Oil Red O on the same day.

### Lipid measurements and tissue lipid staining

Biochemical measurement of kidney cortex triglycerides and Oil Red O lipid staining were performed as previously described [17]. Briefly, frozen tissue samples were homogenized using a Polytron (Brinkmann Instruments, Westbury, NY) in 300 mM mannitol, 18 mM HEPES and 5 mM EGTA (pH 7.5), and lipids were extracted by the method of Folch *et al.* [28]. Total triglyceride content was quantified using a triglyceride colorimetric assay kit (Sigma, St. Louis, MO) according to the method of Danno *et al.* [29]. Measurements from each tissue sample were performed in triplicate, with mean triglyceride values for each subject included in further analyses.

For lipid staining, 4 µm tissue sections were rinsed with distilled deionized water, rinsed briefly with 60% isopropanol, stained with Oil Red O for 1 hour, and then subjected to standard hematoxylin staining and mounted on glass slides. Quantitative assessment of Oil Red O staining was performed in a blinded fashion by two independent investigators using the color deconvolution method of Ruifrock and Johnston [30] and the National Institutes of Health ImageJ software [31].

Tissue ceramides were measured in frozen tissue samples by the Mouse Metabolic Phenotyping Core at UT Southwestern Medical Center using high performance liquid chromatography-electrospray ionization-tandem mass spectrometry (HPLC-ESI-MS/MS), on a Shimadzu Prominence HPLC system coupled to an API 5000 LC-MS/MS mass spectrometer (ABSCIEX, Framingham, MA) equipped with a Turbo VTM electrospray ionization source operated in positive mode. Quantitative analysis of ceramides was achieved using selective reaction monitoring scan mode, with the concentration of each analyte determined according to calibration curves using peak-area ratio of analyte vs. corresponding internal standard. Calibration curves were generated using serial dilutions of ceramide standards (Avanti Polar Lipids, Alabaster, AL).

### Animals

Nine Zucker ZDF (fa/fa) rats, 7–10 months of age, and eight age-matched lean wild-type (+/+) rats, were a gift from Dr. Roger Unger (Touchstone Center for Diabetes Research, UT Southwestern Medical Center). Rats were fed standard chow (Harlan Teklad, Madison, WI) ad libitum and had free access to water. Euthanasia was performed by exsanguination under anesthesia with ketamine-xylazine-acepromazine (100, 10, and 1 mg/kg intraperitoneally). Kidneys were dissected on ice, and cortical samples were frozen in liquid nitrogen and stored at −80°C for lipid measurements.

### Statistical analysis

For continuous variables, one-way analysis of variance with orthogonal linear contrasts was used to assess the trend over the kidney cortex triglyceride content tertiles. Adjustments for potential confounding variables were made with analysis of covariance models. Categorical variables were analyzed with the Cochran-Armitage trend test. Correlations were evaluated with Spearman rank-order correlation coefficients. A two-sided P value<0.05 was considered statistically significant. Analyses were performed with SAS v9.3 (SAS Institute, Cary, NC).

## Results

### Kidney cortex triglyceride content and characteristics of the study population

**Figure 1:**
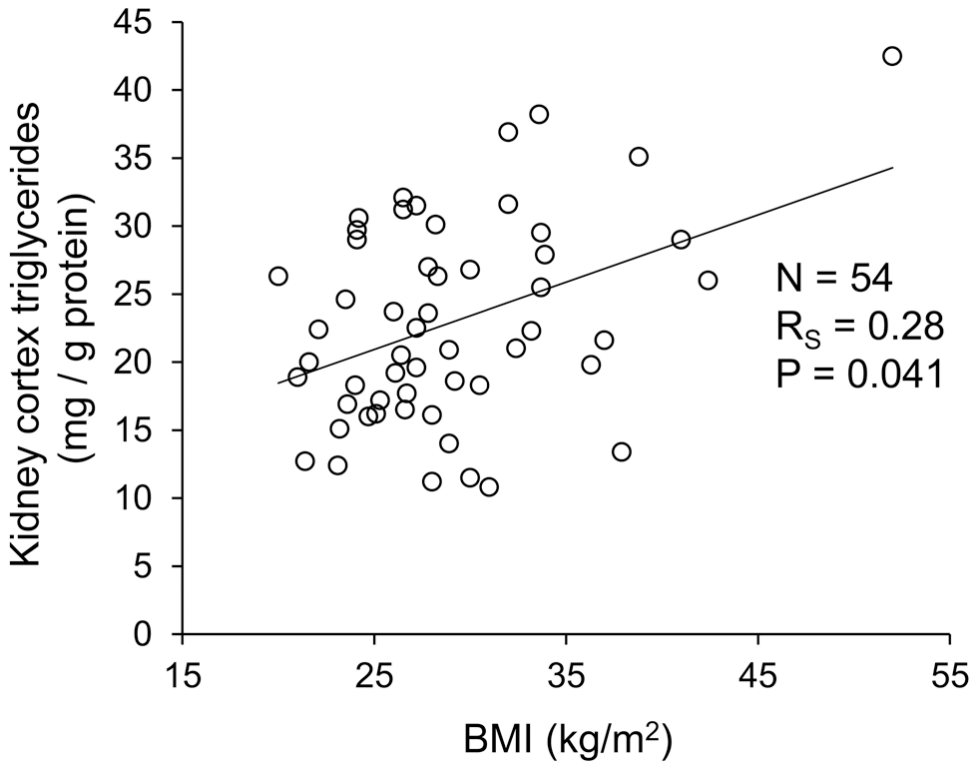
Kidney cortex triglyceride content and relationship with body mass index (BMI). Nephrectomy cortex samples were obtained from 54 patients with serum creatinine <1.5 mg/dL and no proteinuria. All surgical specimens were dissected by experienced clinical pathologists and confirmed to be healthy, away from the tumor. R_S_, Spearman rank-order correlation coefficient.

Triglyceride content was measured biochemically in non-cancerous kidney cortex samples obtained from 54 patients undergoing total nephrectomy. Selected demographic characteristics of the study population are presented in **Table 1**, with further stratification by tertiles of cortical triglyceride content. Body mass index (BMI) increased across tertiles of increasing kidney cortex triglyceride, and there was a significant positive correlation between triglycerides and BMI analyzed as continuous variables (**Figure 1**). Patients with higher renal cortical triglyceride content tended to be younger in the unadjusted analysis, but only BMI was a significant predictor of kidney triglyceride content (P = 0.003) in a multivariable analysis including age and BMI as covariates. There were also no statistically significant differences in tertile distribution by gender or race/ethnicity, though women and minorities were numerically over-represented in the highest tertile (**Table 1**).

**Table 1:** Demographic and anthropometric characteristics.

	Overall cohort (N = 54)	Tertile 1 (N = 18)	Tertile 2 (N = 19)	Tertile 3 (N = 17)	P_trend_	P for tertile 1 vs. 3
**Body mass index**,* kg/m^2^	27.8 (24.9, 32.2)	26.7 (24.2, 29.1)	27.2 (24.8, 32.8)	30.0 (26.5, 33.7)	0.06	0.04
**Age**	65 (56, 71)	66 (60, 71)	66 (58, 74)	59 (50, 64)	0.07	0.06
**Gender**					0.41	0.41
Female	19 (35.2%)	6 (33.3%)	5 (26.3%)	8 (47.1%)		
Male	35 (64.8%)	12 (66.7%)	14 (73.7%)	9 (52.9%)		
**Race/ethnicity** †					0.61	0.63
Black	4 (7.4%)	1 (5.6%)	0	3 (17.6%)		
Hispanic	3 (5.6%)	1 (5.6%)	1 (5.3%)	1 (5.9%)		
Native American	1 (1.9%)	0	0	1 (5.9%)		
White	43 (79.6%)	14 (77.8%)	17 (89.5%)	12 (70.6%)		
Unknown	3 (5.6%)	2 (11.1%)	1 (5.3%)	0		

Data are presented as median (25^th^, 75^th^ percentiles) or number of subjects (percentage) for the overall study population, as well as stratified by tertiles of renal cortical triglyceride content.

* Adjusted for body mass index.

† Because of rounding, not all percentages for race/ethnicity add up to exactly 100%.

Serum creatinine, estimated glomerular filtration rate (eGFR), serum lipids (with and without adjustment for the use of antidyslipidemic medications) and diagnosed hypertension were not different across tertiles of kidney cortex triglyceride content (**Table 2**). We noted a borderline significant association between higher renal cortical triglyceride content and documented type 2 diabetes in unadjusted analyses, but statistical significance was not upheld after adjustment for BMI.

**Table 2:** Biochemical and clinical characteristics.

	Overall cohort	Tertile 1	Tertile 2	Tertile 3	P_trend_	P for tertile 1 vs. 3
**Creatinine** (mg/dL)	1.0 (0.8, 1.2)	1.0 (0.8, 1.2)	1.0 (0.9, 1.1)	0.9 (0.7, 1.3)	0.62	0.82
**eGFR** * (ml/min/1.73 m^2^)	79 (62, 92)	74 (61, 98)	81 (67, 90)	75 (61, 88)	0.64	0.85
**Triglycerides** (mg/dL)†	132 (96, 195)	132 (95, 150)	154 (93, 232)	125 (101, 234)	0.47	0.52
**Total Cholesterol** (mg/dL)†	171 (137, 200)	190 (144, 194)	180 (135, 214)	153 (135, 179)	0.35	0.26
**HDL-Cholesterol** (mg/dL)†	39 (34, 49)	44 (34, 51)	36 (31, 45)	39 (35, 47)	0.61	0.56
**LDL-Cholesterol** (mg/dL)†	95 (76, 117)	106 (90, 119)	97 (79, 128)	85 (65, 96)	0.15 (0.58§)	0.10 (0.24§)
**Antidyslipidemic medications** ‡	13 (31.7%)	2 (18.2%)	6 (35.3%)	5 (29.4%)	0.30	0.28
**Diabetes** ∥	12 (22.2%)	1 (5.6%)	6 (31.6%)	5 (29.4%)	0.09 (0.24#)	0.06 (0.12#)
**Hypertension**	35 (64.8%)	14 (77.8%)	11 (57.9%)	10 (58.8%)	0.24	0.23

Data are presented as median (25^th^, 75^th^ percentiles) or number of subjects (percentage) for the overall study population, as well as stratified by tertiles of renal cortical triglyceride content.

* eGFR was calculated using the CKD-EPI formula, http://www.kidney.org/professionals/kdoqi/gfr_calculator.cfm.

† Lipid values were available for 34 of the 54 study participants (13, 10 and 11 respectively in the 3 tertiles).

‡ Antidyslipidemic medications included statins in 11 patients, statin combined with cholesterol absorption inhibitor in 1 patient, and fibrate in 1 patient.

§ Adjusted for the use of antidyslipidemic medications.

∥ All type 2 diabetes.

Of these, 4 were taking metformin, 3 sulfonylurea (glipizide or glyburide), one metformin+glimepiride, and 4 were managed with lifestyle intervention alone.

# Adjusted for body mass index.

### Localization of triglyceride droplets

**Figure 2:**
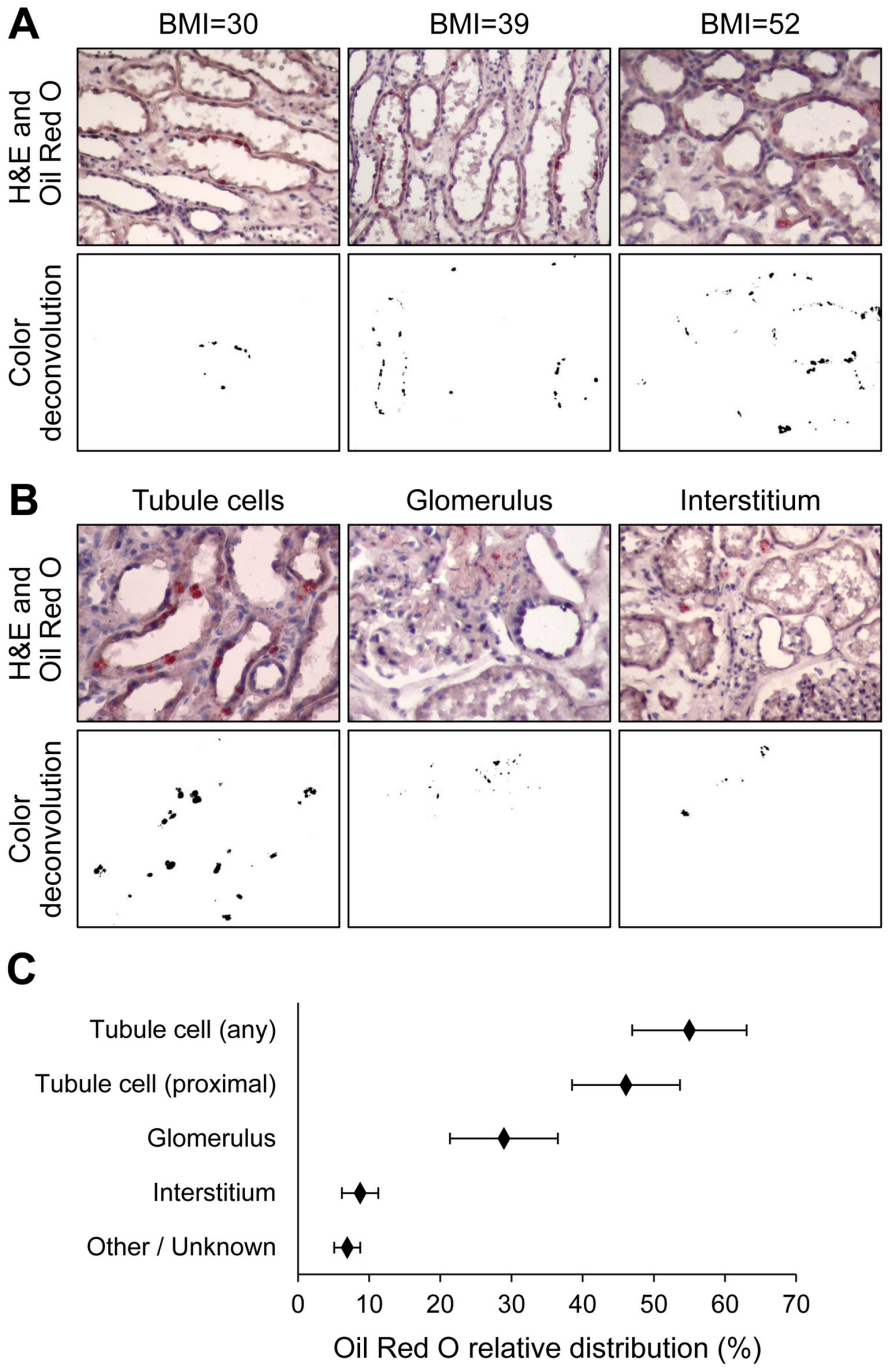
Lipid staining. Kidney cortex sections were stained with Oil Red O and standard hematoxylin to visualize lipid localization within renal structures. After image acquisition, a computer-based color deconvolution and thresholding algorithm was used to separately visualize and quantify Oil Red O staining, with the original image serving as reference for the manual assignment of lipid staining to discrete structures (i.e. tubule cells, glomeruli, interstitium, other). A. Oil Red O staining was noted predominantly in proximal tubule cells, and generally increased with increasing body mass index (BMI). B. Examples of the localization of lipids within tubule cells, glomeruli and interstitium. C. Relative distribution of lipids within kidney structures based on Oil Red O staining quantified by color deconvolution in 29 study participants with available fixed tissue. Whiskers represent 95% confidence intervals.

Triglyceride localization within kidney structures was examined in a blinded fashion by two investigators using Oil Red O staining of lipid droplets followed by computerized color deconvolution, in a subset of 29 subjects for which fixed tissue was available. Of these, 19 subjects had detectable Oil Red O staining. In spite of inter-individual variability, generally more lipid droplets were detected in samples from individuals with higher BMI (**Figure 2A**). Lipid droplets were predominantly localized in tubular cells, mostly in proximal tubules, and to a lesser extent within glomeruli and in the interstitium (**Figures 2B and 2C**). Of note, even in samples with high triglyceride content by biochemistry, lipid droplets detectable by Oil Red O staining and optical microscopy had a relatively sparse distribution, likely because only larger droplets can be visualized using this technique [32].

### Kidney cortex ceramide content

**Figure 3:**
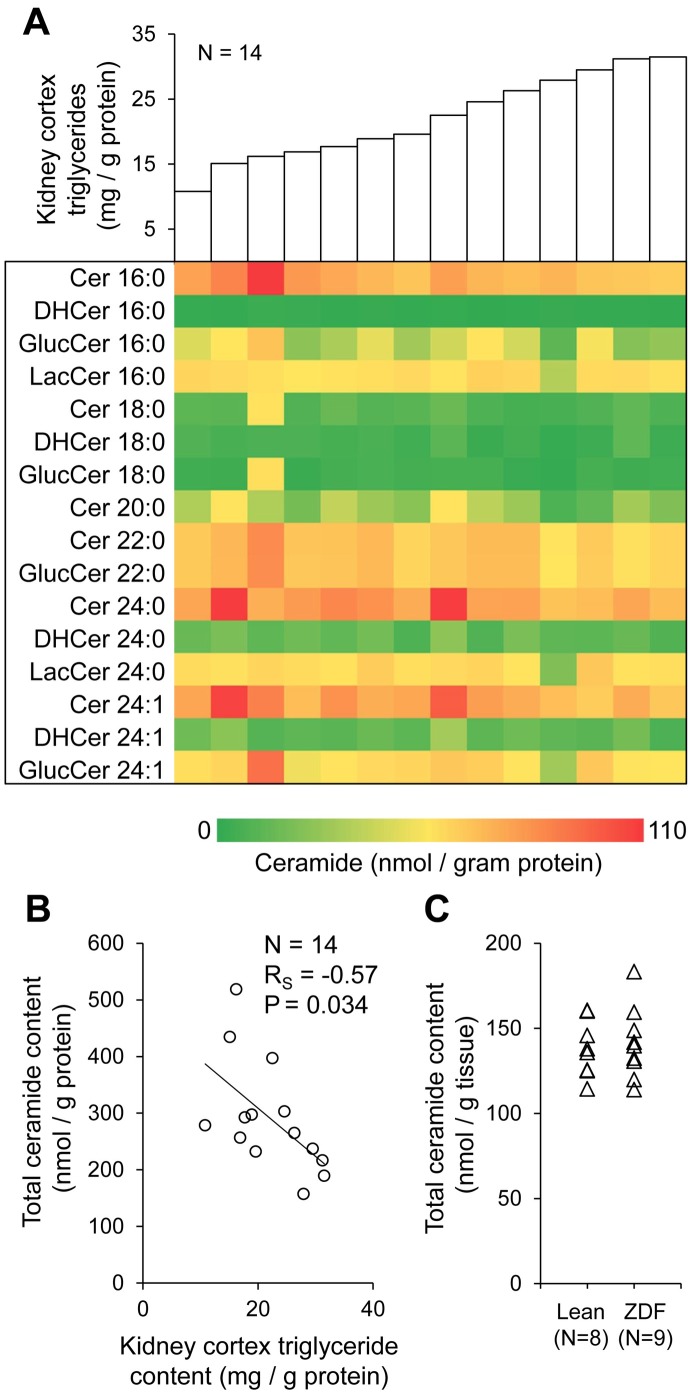
Kidney cortex ceramide content and relationship with triglycerides. Sixteen common ceramide species (Cer, ceramide; GlucCer, glucosylceramide; LacCer, lactosylceramide; DHCer, dihydroceramide) were measured by high performance liquid chromatography-electrospray ionization-tandem mass spectrometry (HPLC-ESI-MS/MS) in kidney cortex samples previously frozen at −80°C. A. The abundance of each ceramide species in human kidney cortex samples with increasing triglyceride content. B. Relationship between total ceramide content (molar sum of all measured ceramides) and triglyceride content in human kidney cortex samples. R_S_, Spearman rank-order correlation coefficient. C. Total ceramide content in kidney cortex samples from Zucker diabetic fatty (ZDF) and lean control rats.

Kidney cortex ceramide content was measured retrospectively in samples from a subset of 14 patients with available tissue stored at −80°C. The abundance of 16 common ceramide species in individual study subjects is shown in **Figure 3A**. We noted an inverse correlation trend between tissue triglycerides and several ceramides, with ceramide 16:0 reaching statistical significance (R = −0.61, P = 0.02). As shown in **Figure 3B**, within-sample molar sums of all measured ceramides were significantly and inversely correlated with triglyceride content. In spite of the positive correlation between triglycerides and BMI in the overall study population, there was no detectable relationship between BMI and individual ceramides or total ceramide content in the subset of subjects with measured renal ceramide content.

In an effort to further explore the significance of ceramides in relationship with renal lipid accumulation, we also measured ceramide content in obese Zucker diabetic fatty (ZDF) rats and lean age-matched controls. In spite of much higher triglyceride content in the kidney cortex of ZDF versus lean rats, there was no difference in total ceramides between groups (**Figure 3C**), and no relationship between total ceramides and triglyceride content.

## Discussion

### Key findings and interpretation

There are several key findings from this study of normal kidney surgical specimens obtained from 54 patients undergoing nephrectomy. There was a modest but statistically robust correlation between body mass index and total kidney cortex triglyceride content, suggesting that renal lipid accumulation occurs in humans with increasing body size. Lipid droplets detectable by conventional staining were primarily localized within epithelial cells of the proximal tubule, and to a lesser extent within glomeruli, suggesting that lipid accumulation and lipotoxicity may interfere with the normal physiology of these structures. Finally, evaluation of kidney ceramides in humans and rodents suggested that lipid accumulation in the kidney may not associate with lipotoxicity via the ceramide pathway.

### Obesity and triglyceride accumulation in non-adipose tissues

Lipid accumulation with increasing BMI has been described in multiple non-adipose tissues, including the liver [21], [23], pancreas [22], myocardium [23] and skeletal muscle [20]. With some exceptions, such as the “athlete's paradox” of high intramuscular lipid associated with marked insulin sensitivity in endurance-trained athletes [33], lipid accumulation has been associated with lipotoxicity and organ dysfunction [24]–[27].

In the human kidney, various patterns of lipid accumulation have been described in patients with genetic defects of lipid metabolism, including familial dysbetalipoproteinemia [34], lecithin-cholesterol acyltransferase deficiency [35], lipoprotein glomerulopathy [36] and alpha-galactosidase A deficiency (Fabry's disease) [37], as well as in patients with acquired conditions such as hypertensive nephrosclerosis [38], focal segmental glomerulosclerosis [39], minimal change disease with massive proteinuria [40] and hepatorenal syndrome [41]. However, whether lipid accumulation occurs with increasing BMI was not known prior to the present study.

As the general definition of steatosis is “any abnormal accumulation of triglycerides within parenchymal cells” [42], our data suggest that obesity may be associated with renal steatosis, although absolute triglyceride levels in the steatotic kidney are much lower than in the steatotic liver [43].

### Localization of lipid droplets and pathophysiological implications

A large number of animal studies using genetic, pharmacologic or dietary manipulations have shown that renal lipid accumulation can occur both in glomeruli and in tubule cells, primarily in proximal tubules, with some variations between genetic strains and experimental models (reviewed in [9]). Lipid accumulation in glomeruli as well as in tubule cells has been postulated to contribute to the pathogenesis of kidney disease in animal models of progressive renal injury [8], [9]. In addition, prior *in vivo* and *in vitro* studies from our group have proposed a causal relationship between lipid accumulation in the proximal tubule and altered renal acidification, which can increase uric acid stone risk [17], [18]. It is absolutely imperative to determine whether these studies are relevant for human pathophysiology. One *sine qua non* condition is detectability of lipid accumulation within the respective renal structures in at-risk individuals. Our findings that kidney cortex triglycerides are localized in both tubule cells and in glomeruli, and track with increasing BMI, suggest that renal lipid accumulation could play a role in obesity-related kidney disease and nephrolithiasis risk.

### Lipid droplets, lipotoxicity and the role of ceramides

Intracellular lipid droplets are not static fat depots, but dynamic and highly regulated organelles with important normal functions in cellular energy storage and metabolism [44]. When net delivery of fatty acids to non-adipose cells exceeds cellular energy needs or beta-oxidative capacity, one key mechanism of defense against lipotoxicity is the storage of excess fatty acids as triglycerides in lipid droplets. It is thus important to note that triglyceride accumulation is not considered harmful *per se*, but is instead a quantifiable marker of a disturbed balance between fatty acid supply and utilization [8], [9], [45]. Excess fatty acids that are not beta-oxidized or incorporated in triglycerides enter alternative metabolic pathways, resulting in increased cellular content of potentially lipotoxic metabolites, with ceramides as a much touted candidate [8], [9], [45]. This model is compatible with the negative correlation between renal triglyceride and ceramide levels in a subset of samples from our study, suggesting that effective incorporation of excess fatty acids into triglycerides may protect against ceramide-induced renal lipotoxicity.

Importantly, there was no detectable relationship between ceramide levels and BMI in our dataset. We postulate that our data on triglycerides reflect a general state of obesity-associated renal fatty acid oversupply, while our data on ceramides reflect inter-individual differences in the metabolic fates of excess fatty acids in the kidney due to factors unrelated to BMI. These differences could potentially contribute to clinical variability, with obesity-related kidney disease and nephrolithiasis risk not uniformly manifested in the general population. However, while ceramides have been implicated in lipotoxicity in other organs, little is known about their role in the kidney, and the potential contribution of other lipid metabolites to renal lipotoxicity is also unclear [8], [24], [46], [47].

### Renal ceramides in Zucker diabetic fatty rats

To further explore the potential role of ceramides in renal pathophysiology, while controlling for the genetic and environmental heterogeneity inherent in human subject research, we studied a rodent model in which renal triglyceride accumulation has been linked with surrogate functional markers of uric acid stone risk, including data showing that reduction of triglyceride accumulation with a PPARγ agonist reversed the functional defects [18], [19]. There were no differences in total kidney cortex ceramide content between ZDF rats and lean control rats, suggesting that renal dysfunction in ZDF rats is not attributable to ceramide-induced lipotoxicity. Other lipid metabolites may contribute to renal lipotoxicity in this animal model [8], [24], [46], [47].

### Strengths and Limitations

Key strengths of the present study include the quantitation of triglycerides by a combination of direct biochemical and histological methods and the assessment of a relatively large number of samples from patients with a wide range of BMI. While most recent studies in humans have evaluated triglyceride content in various tissues indirectly by ^1^H magnetic resonance spectroscopy (MRS) [20]–[23], direct biochemical quantitation of triglycerides, as used in the present study, remains the gold-standard [48]. Despite widespread use in the heart, skeletal muscle, liver and pancreas [49], MRS-based measurement of triglycerides in the kidney has only recently been reported by Hammer *et al.* [50]. However, the technique does not have adequate reliability in our hands. We and our collaborators have performed careful measurements including respiratory motion compensation, but these yielded borderline signal-to-noise ratios and unacceptable intra-assay variation (unpublished data). This is largely attributable to the much lower fat content in the kidney, even in pathologic states, compared with other tissues in which this technique is established. The study of Hammer *et al.* found mean triglyceride-to-water peak ratios in the renal parenchyma less than 0.5%, with a reported intra-assay coefficient of variation (CV) of 27% in optimal conditions [50]. For comparison, triglyceride peak ratios as high as 40% have been reported in steatotic liver, with CV = 8.5% [51].

The study also has important limitations. Our cohort of patients undergoing nephrectomy for renal tumors is not representative of the general population. Measures of adiposity other than BMI, such as waist circumference, waist to hip ratio, or truncal fat content determined by dual energy X-ray absorptiometry, were not available. Finally, because of limited tissue availability, we could only perform Oil Red O staining and ceramide measurements in subsets of the study population.

## Conclusions

This is the first demonstration of obesity-related triglyceride accumulation (steatosis) in the human kidney. Even though the amount of lipid is not large and the distribution is sporadic, the predominant localization of lipid droplets appears to be within proximal tubule cells, and to a lesser extent within glomeruli. Triglyceride accumulation in individuals with high BMI is likely a marker of renal fatty acid oversupply, as well as a cellular defense mechanism against the accumulation of potentially toxic metabolites, as suggested by our quantitation of 16 common ceramide species. This study supports the potential association between renal lipid accumulation and obesity-related kidney disease and nephrolithiasis risk.
